# Recruitment, retention and employment growth in the long-term care sector in England

**DOI:** 10.3389/fpubh.2022.969098

**Published:** 2022-10-28

**Authors:** Hansel Teo, Florin Vadean, Eirini-Christina Saloniki

**Affiliations:** ^1^Personal Social Services Research Unit (PSSRU), University of Kent, Canterbury, United Kingdom; ^2^Department of Applied Health Research, University College London, London, United Kingdom; ^3^National Institute for Health and Care Research (NIHR) Applied Research Collaboration North Thames, London, United Kingdom

**Keywords:** long-term care, recruitment, retention, employment growth, England, turnover, hiring

## Abstract

This paper studies the relationship between turnover, hiring and employment growth in the long-term care (LTC) sector in England and sheds light on how challenges in both recruitment and retention affect the sector's ability to meet growing demand for care services. Using the Adult Social Care Workforce Data Set (ASC-WDS), a large longitudinal dataset of LTC establishments in England, and fixed effects estimation methods we: (a) quantify the relationship between the in/outflow of care workers and the expansion/contraction of employment within establishments, (b) establish the role of staff retention policy for workforce expansion, and (c) identify the role of recruitment frictions and its impact on hiring and employment contraction. Our analysis indicates that care worker turnover and employment growth are negatively related. A one percentage point increase in employment contraction is associated with a 0.71 percentage point rise in turnover, while a one percentage point increase in employment expansion is associated with a 0.23 percentage point fall in turnover. In contrast, we find that hiring rates and employment growth are positively related. A one percentage point increase in employment expansion is associated with a 0.76 percentage point rise in hiring, while a one percentage point increase in employment contraction is associated with a 0.26 percentage point decrease in hiring. We argue that the negative turnover-employment growth relationship within expanding establishments provides evidence that better staff retention is associated with higher employment growth. Using information on establishments' annual change in vacancies, and controlling for changes in new labor demand, we also find rising year-on-year vacancies amongst establishments with declining employment. This provides evidence that recruitment frictions drive the declining rate of replacement hiring amongst contracting establishments. Across sectors, we find that the employment growth-turnover and the employment decline-hiring relationships are relatively stronger in the private and voluntary sectors compared to the public sector, suggesting that the impact of staff retention and recruitment frictions on employment is more acute in these sectors.

## Introduction

Long-term demographic trends (i.e., an aging society and the increase in life expectancy of people with disabilities) in many developed countries imply that the demand for long-term care (LTC) services will continue to increase. A key input for providing these services is labor. In England, around 1.7 million people work in the adult LTC sector (1). Nevertheless, recent estimates suggest that this workforce needs to grow by an additional 29 per cent (490,000 jobs) by 2035 in order to keep up with the increasing demand (1). Within this wider context, the LTC sector in England faces significant workforce-related challenges in keeping up with the increased demand pressure.

Two key challenges are workforce recruitment and retention. The size of the LTC workforce at any point in time reflects the outcome of continuous inflows and outflows of workers. Existing studies on LTC workforce issues have focussed relatively more on outflows, in particular, on measures of staff turnover and retention rates. Staff turnover rates capture the share of existing workers leaving an employer over a given period while retention rates capture an employer's ability to retain the same staff (2). Studies of turnover rates in the LTC sector have provided valuable insights and highlighted their association with factors such as ownership structure (3–5), management style (6, 7), job satisfaction and employment conditions (8, 9). Related work has also found that staff turnover is associated with quality of care measures (2, 10–14). Nevertheless, turnover forms only part of the picture because the impact of staff turnover on the care workforce is mediated by care providers' ability to replace leavers through recruitment.

Recruitment in the LTC sector has received less attention. This partly reflects the fact that recruitment information is not typically available in the survey datasets used to study the LTC workforce—e.g., the Ohio Biennial Survey of LTC facilities (4, 5) and survey of nursing home administrators (2, 15). In addition, unlike turnover and retention rates, there seems to be less consensus on appropriate measures for capturing difficulties in staff recruitment. Most existing studies typically focus on vacancy rates, which measure the share of unfilled positions as a share of total filled and unfilled positions at a point in time. Except for a slight decrease between 2019 and 2021, vacancy rates in the LTC sector in England have increased steadily from 4.4 to 7.5 per cent between 2013 and 2019 (1). Furthermore, vacancy rates in the LTC sector are high relative to the U.K. economy-wide vacancy rate (2.1 per cent). These statistics suggest that the LTC sector faces exceptional challenges in meeting its workforce requirements and that these problems have been persistent and growing over time.

Broadly, low pay levels (often at minimum wage), lack of status (as care work is not recognized as a profession), and limited opportunities for career progression have been identified as factors contributing to recruitment difficulties (16–19). At a more granular level, studies have found differences in vacancy rates across care settings (1, 20) and geographies (20, 21). Moreover, vacancy rates have been found to be increasing with the share of employees on zero-hours contracts and the average days of sick leave per employee (9). Nonetheless, job vacancy rates provide only a snapshot of the number of unfilled positions and do not inform about the extent of deeper recruitment issues.

This paper studies the relationship between turnover, hiring and employment growth and their implications for the LTC sector's ability to maintain a care workforce able to meet rising demand. To do so, we proceed in three steps. First, we use longitudinal data on LTC establishments and workers in England and panel fixed effect regression methods to quantify the relationship between the in/outflow of care workers and the expansion/contraction of employment at LTC establishments. Second, focusing on establishments with expanding employment, we analyze the relative contributions of staff inflow (i.e., hiring) and outflow (i.e., turnover) to employment growth and establish the role of staff retention in workforce expansion. Third, focusing on establishments with contracting employment, we analyze year-on-year changes in establishments' vacancies and identify the role of recruitment frictions and its impact on hiring and employment contraction.

We find a negative relationship between care worker turnover and employment growth along the growth distribution. Our estimates imply that a one percentage point increase in employment decline is associated with a 0.71 percentage point rise in turnover rate, while a one percentage point increase in employment growth is associated with a 0.23 percentage point fall in turnover rate. In other words, establishments that are contracting (expanding) more rapidly have a higher (lower) share of workers leaving.

Turning to hiring, we find a positive relationship between hiring rates and employment growth along the growth distribution. Our estimates imply that a one percentage point increase in annual care worker employment is associated with a 0.76 percentage point increase in hiring, while a one percentage point decrease in year-on-year employment is associated with a 0.26 percentage point decrease in hiring. That is, establishments that are growing more rapidly tend to hire new care workers at a faster rate and establishments that are contracting more rapidly tend to hire replacements at a lower rate.

We argue that our findings suggest that staff retention policies (i.e., measures which reduce the rate at which staff leave an establishment) are important for expanding employment. Intuitively, an establishment's care workforce can expand due to a combination of more rapid hiring or reduced staff turnover. If staff retention policy were indeed irrelevant for employment growth, then we would expect turnover to be constant or even increasing as establishments expand more rapidly, and hiring to increase at least one-for-one with employment growth. Instead, we find that more rapid employment growth is systematically associated with reduced staff turnover and a less than one-for-one increase in hiring rates. Together, these suggest that employment expansion in the LTC sector, in general, involves both increasing the inflow of workers through hiring and moderating the outflow of workers through better staff retention.

To understand why establishments with contracting employment have lower rates of hiring, we use a new measure, namely the annual change in vacancies, along with information on employment and care service utilization to isolate the effect of frictions in recruitment. In this way, we can test whether the decrease in hiring amongst contracting establishments reflects intentional downsizing policies or if difficulties in recruitment are instead the key contributor. Intuitively, since each new vacancy represents effort to fill an unfilled position, if contracting employers were indeed intentionally reducing hiring to downsize, then, after controlling for changes in labor demand, we should not see a year-on-year increase in vacancies. We find that amongst establishments with contracting employment, a one percentage point increase in employment decline is associated with an increase of 1.15 unfilled vacancies. This finding contradicts the competing claim that the employment declines we observe are purely due to intentional downsizing.

## Materials and methods

### Data

We used data from the Adult Social Care Workforce Data Set (ASC-WDS), the leading source of LTC workforce intelligence in England. The dataset is managed by Skills for Care and includes information on over 20,000 LTC establishments and over 700,000 workers, covering about 50 per cent of the LTC market. The information is rich at both establishment (e.g., type of service provided, sector, establishment size, count of employees and job roles, starters, leavers and vacancies, etc.) and worker level (e.g., age, gender, nationality, qualifications, pay, working hours, job role and job type). Public LTC employers update their data on a mandatory basis in September each year. Independent employers submit data on a voluntary basis, but are incentivised to do so by access to workforce development grants. All data in the ASC-WDS have been updated or confirmed to be up to date within the last 2 years, and about 80 per cent of employers have updated their data in the past 6 months. Although the dataset does not cover all independent sector establishments, it does have a large enough sample to provide a solid basis for reliable workforce estimates at both national and local level. All ASC-WDS data have been validated at source and have undergone rigorous quality checks (1, 22).

We used data from four cuts of the ASC-WDS: October 2016, October 2017, October 2018, and October 2019, matched at establishment level, and with some variables generated from the worker dataset (e.g., mean age, mean female rate, mean hourly wage, share of staff on zero-hours contracts, etc.). Skills for Care assigns to each establishment a unique and permanent ID. We excluded establishments who did not update their records for more than 6 months. We kept establishments providing either care home services (with or without nursing) or domiciliary care to adults (i.e., service users aged 18 and over). Public sector (i.e., statutory local authority), private (i.e., for-profit), and voluntary (i.e., not-for-profit) sector providers were all included. In addition, we restricted our sample to establishments present in the dataset for at least 2 consecutive years, and which reported having employed care workers on a permanent or temporary contract in at least the first year. After excluding observations with missing values for required variables, the resulting unbalanced panel contains 10,773 establishment-year observations corresponding to 4,199 unique establishments.

Due to the sample selection criteria, we do not expect our analysis sample to be fully representative of the English LTC sector. For example, the need for consecutive observations precludes start-ups in 2019 and closures in 2016. Furthermore, we would also expect public sector establishments to be over-represented due to the sampling structure of the ASC-WDS. To gauge the representativeness of our analysis sample, we computed sampling weights for each observation. These weights, calibrated by the raking procedure, target care setting × year specific totals obtained from the Care Quality Commission (CQC) directory. Table A2 compares the summary statistics of the variables in our model based on weighted and unweighted data. Overall, this method suggests that our sample over-represents residential care establishments, public sector establishments and those with “Good/Outstanding” CQC quality ratings. Despite these differences, robustness checks show that our main results remain largely unaffected after accounting for sample representativeness through weighting.

### Worker flow rates, employment growth rates and vacancies

The ASC-WDS contains, for each job role, information on the stock of permanent and temporary staff, the number of staff that left, and the number of staff that started work in the previous 12 months. We define an establishment's annual care worker turnover rate at time *t* as the reported number of care workers that left the establishment divided by the average stock of care workers employed at *t*−1 and *t*. Similarly, we define an establishment's annual care worker hiring rate at time *t* as the reported number of starters divided by the average stock of care workers employed at *t*−1 and *t*. Finally, we define an establishment's annual care worker employment growth rate as the difference in the stock of care workers between *t*−1 and *t* divided by the average number of care workers employed at these two times. The approach of normalizing flow rates by the average employment between two time points follows the literature on job and worker flows (23–26). The ASC-WDS also contains information on the number of staff vacancies for care worker roles at the time of the last update, and we use this to define the establishment's annual change in care worker vacancies, i.e., as the reported number of vacancies at *t* minus the reported number at *t* − 1.

### Econometric model

We assume that establishment-level worker flows are related to employment growth via the following additive-linear form:


(1) $$y_{it}=\;\gamma (g_{it})+x_{it}^{{}^{\prime }}\beta +\;\alpha_{i}+\delta_{t}+\;\varepsilon_{it}$$

where the dependent variable, *y*_*it*_, is either the turnover rate or hiring rate of establishment *i* in year *t*, *α*_*i*_ represents time-invariant unobserved establishment-level heterogeneity, *δ*_*t*_ aggregate time effects, *x*_*it*_ is a set of non-growth explanatory variables and *ε*_*it*_ is an idiosyncratic error term. *g*_*it*_ is the growth rate of employment in year *t* while the function *γ*(*g*) describes the relationship between employment growth and worker flows. The latter relationship could be non-linear and non-monotonic in general. The objective of our statistical analysis is to quantify the relationship between job and worker flows as encapsulated in *γ*(*g*).

Early studies have used parametric specifications of Equation (1) to examine the relationship between job and worker flows in the Danish manufacturing sector (27) and the U.S. (28). More recent studies also estimated non-parametric versions of this specification in a variety of contexts (24, 25, 29, 30). For our descriptive analysis, we allow for non-parametric relationships between job and worker flows. To enable a more straightforward interpretation, our regression analysis will use a simple piecewise linear form. Specifically, let *I*(*g*_*it*_ > 0) be an indicator for strictly positive employment growth and *I*(*g*_*it*_ < 0) for strictly negative growth. We estimate the following model using fixed-effects panel regression.


$$\begin{array}{l} y_{it}=\;\gamma^{+}g_{it}•I\left(g_{it}>0\right)+\gamma^{-}g_{it}•I\left(g_{it}<0\right)+x_{it}^{{}^{\prime }}\beta \end{array}$$

(2) $$\begin{array}{l} +\;\alpha_{i}+\delta_{t}+\;\varepsilon_{it} \end{array}$$

Equation (2) allows for the relationship between growth and worker flows to differ between expanding and contracting establishments but assumes that the relationship is linear within each group. This modeling choice balances flexibility against interpretability of the resulting regression estimates and is broadly consistent with descriptive graphical representations of the data. Nonetheless, we assess the sensitivity of our findings with respect to this functional form assumption in our robustness analysis.

*x*_*it*_ contains a rich set of time-varying covariates. These include dummy variable interactions for care setting and year, sector and year and local area and year. Year dummies, *δ*_*t*_, capture aggregate socio-economic changes that affect hiring and separations, while the care setting × year and sector × year interactions capture policy and sectoral shifts. We define a local area to be one of the 150 Councils with Adult Social Services Responsibilities (CASSRs) in England and include local area × year interactions to capture the heterogeneity of local labor markets in England. Our full covariate specification also includes: the overall quality rating by the CQC (England's independent health and LTC regulator), local market conditions (unemployment rate, mean hourly wage in the lowest quartile, average house prices, care home competition index), establishment size and staffing (total employment and the ratio of direct care workers to service users), remuneration policies (mean hourly wage of care workers and share of workers on zero-hours contracts), type of care users (dummies for dementia and mental disorders), staff training (share of workers who have completed dementia care and Dignity, Respect & Person Centred care training). Detailed definitions of the variables used in our model are listed in Table A1.

Following existing work on the LTC workforce which has found that the stability of the direct care workforce in an establishment is positively correlated with the stability of its managerial staff (4, 15, 21), we further account for managerial staff stability in the form of managerial turnover in our regression model. In this regard, contemporaneous managerial turnover is likely to be endogenous since both care worker and manager separations over a given period could be driven by an unobserved establishment-level shock, such as organizational restructuring. As such, we instead use 1 year lagged managerial turnover rates as a proxy for managerial staff stability. Because turnover at reporting time *t* captures the number of leavers between *t*−1 and *t*, our specification captures how managerial staff stability between *t*−2 and *t*−1 affects care worker separations and hires between *t*−1 and *t*.

## Results

### Descriptive statistics

Table 1 reports the summary statistics of the dependent variables and covariates used in our analysis sample. To examine if there are any systematic differences in these characteristics between establishments with expanding, contracting and stable employment, the last three columns report the mean values of these variables by employment growth category. About 18 per cent of establishment-year observations have zero change in annual care worker employment with the remaining split between contracting (43 per cent) and expanding establishments (39 per cent). Average employment growth rates amongst the latter two groups are very close in magnitude (around −22.0 per cent and +22.3 per cent respectively), resulting in an aggregate employment growth rate close to zero. Turning to establishment-level characteristics, the statistics imply that 2 year average employment is higher amongst expanding and contracting establishments compared to those with stable employment. However, beyond employment levels, there do not appear to be systematic differences in establishment-level characteristics between establishments in the three groups. Finally, the mean values of the dependent variables reported in Table 1 indicate that on average, turnover rates are higher in establishments with declining employment followed by those with increasing employment and then zero growth establishments. In contrast, hiring rates are higher in expanding establishments, followed by contracting establishments and establishments with zero growth. With respect to vacancies, the average annual change is highest amongst establishments with declining employment followed by those with zero growth and then those with increasing employment.

**Table 1:** Estimation sample summary statistics.

	**All care**		**Negative**	**Zero**	**Positive**
	**establishments**		**growth**	**growth**	**growth**
			**establishments**	**establishments**	**establishments**
	**Mean**	**Std Dev**.	**Mean**	**Mean**	**Mean**
Turnover rate of care workers	0.503	0.517	0.637	0.369	0.420
Hiring rate of care workers	0.503	0.547	0.427	0.372	0.646
Annual change in care worker vacancies	0.282	4.491	0.574	0.100	0.048
Care worker employment growth rate	−0.006	0.279	−0.220	0.000	0.223
Establishment with positive employment growth	0.391	–	0.000	0.000	1
Establishment with negative employment growth	0.426	–	1	0.000	0.000
Service utilization growth rate	0.009	0.211	−0.010	0.009	0.031
Two-year average total employment	47.634	47.815	50.804	34.025	50.567
Direct care worker to service user ratio	1.665	4.250	1.603	1.563	1.779
Establishment with service users with dementia	0.554	–	0.565	0.488	0.573
Establishment with service users with mental infirmities	0.643	–	0.643	0.671	0.631
Share of workers completed dementia care training	0.258	–	0.261	0.265	0.252
Share of workers completed DRPC training	0.187	–	0.185	0.191	0.186
Mean age of employees	43.182	4.753	43.343	43.921	42.659
Mean years of experience of employees	8.811	3.893	8.831	9.848	8.302
Mean hourly wage of employed care workers	7.761	0.756	7.780	7.695	7.770
Share of care workers on zero-hours contracts	0.171	–	0.182	0.129	0.179
Turnover rate of managers/supervisors	0.320	0.499	0.349	0.237	0.327
CQC (Overall) rating—Inadequate/Req. improvement	0.135	–	0.139	0.109	0.141
CQC (Overall) rating—Good/Outstanding	0.827	–	0.822	0.849	0.822
CQC (Overall) rating—No rating	0.039	–	0.039	0.042	0.037
Residential care	0.741	–	0.722	0.806	0.730
Domiciliary care	0.259	–	0.278	0.194	0.270
Public sector	0.061	–	0.058	0.054	0.067
Private sector	0.770	–	0.775	0.758	0.771
Voluntary sector	0.169	–	0.167	0.188	0.162
**Observations**	**10,773**		**4,588**	**1,976**	**4,209**

Table 2 reports the mean turnover, hiring and vacancy rates as well as the annual change in vacancies for each year, care setting and sector. Between 2017 and 2019, average turnover rates increased from 46.3 to 55.0 per cent while average hiring rates increased from 47.2 to 53.7 per cent. Average vacancy rates similarly increased from 4.8 per cent in 2017 to 5.8 per cent in 2019. There appears to be a decrease in average annual change in vacancies from 0.47 in 2017 to 0.05 in 2019. Across care settings, domiciliary care establishments face higher average turnover (63.1 per cent vs. 45.8 per cent), hiring (63.7 per cent vs. 45.6 per cent) and vacancy rates (8.4 per cent vs. 4.4 per cent) compared to residential care establishments. Domiciliary care establishments also experience a larger average year-on-year increase in vacancies relative to residential care establishments (0.60 vs. 0.17). Average employment growth is negative and close to zero in both care settings, with domiciliary care establishments showing slightly less contraction than residential care establishments (−0.5 per cent vs. −0.7 per cent).

**Table 2:** Worker flows and vacancies across years, care settings and sectors.

	**Observations**	**Turnover rate**	**Hiring rate**	**Vacancy rate**	**Change in vacancies**	**Employment growth**
Pooled	10,773	0.503	0.503	0.054	0.282	−0.006
2017	3,582	0.463	0.472	0.048	0.474	0.005
2018	4,143	0.504	0.505	0.057	0.275	−0.004
2019	3,048	0.550	0.537	0.058	0.045	−0.023
**Care setting**						
Residential	7,979	0.458	0.456	0.044	0.169	−0.007
Domiciliary	2,794	0.631	0.637	0.084	0.602	−0.005
**Sector**						
Public sector	653	0.201	0.223	0.056	−0.069	0.022
Private sector	8,297	0.556	0.556	0.056	0.316	−0.007
Voluntary sector	1,823	0.372	0.360	0.047	0.254	−0.013

Across sectors, private sector establishments experience higher average turnover and hiring rates (55.6 and 55.6 per cent, respectively) compared to voluntary sector (37.2 and 36.0 per cent, respectively) and public sector establishments (20.1 and 22.3 per cent, respectively). Average vacancy rates across all three sectors are relatively close and range from 4.7 per cent for voluntary sector establishments to 5.6 per cent for public and private sector establishments. Despite their similar vacancy rates, the average annual change in vacancies in the public sector differs quite significantly compared to voluntary and private sectors. While private and voluntary sector establishments experience year-on-year increases of 0.31 and 0.25 vacancies on average, public sector establishments report an average year-on-year decrease in vacancies of 0.07 vacancies. The average employment growth of public sector establishments in our sample is also higher than that of voluntary and private sector establishments (2.2 per cent vs. −0.7 per cent and −1.3 per cent, respectively).

### Staff turnover and employment growth

**Figure 1:**
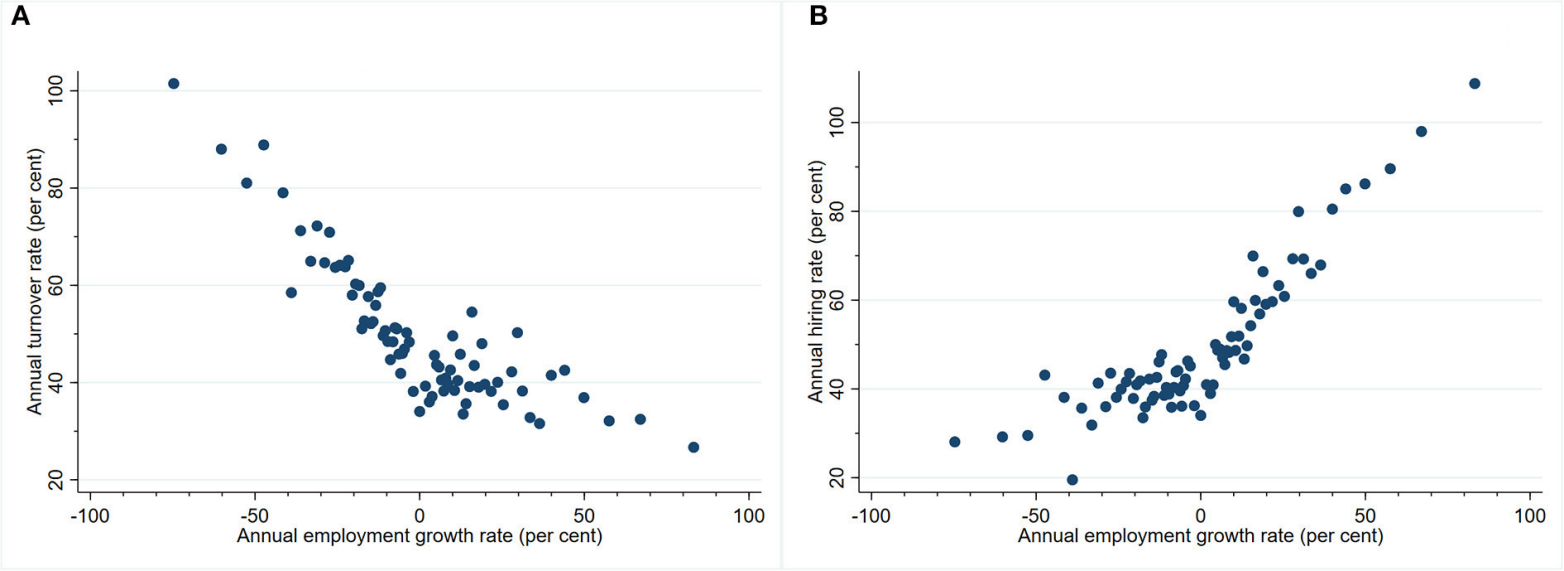
**(A)** Relationship between care worker turnover and employment growth. **(B)** Relationship between care worker hiring and employment growth.

Figure 1A plots the non-parametric relationship between turnover and employment growth in the cross-section. To obtain this figure, establishments in the analysis sample are grouped into 100 equally sized bins based on their employment growth rate. Each point then plots the average turnover rate and employment growth rate of that bin. For presentation purposes, the figure omits outliers corresponding to the top and bottom one per cent employment growth and turnover establishments. The figure shows that care worker separations as a share of employment is decreasing over both negative and positive employment growth regions. Amongst contracting establishments, the turnover rate increases almost linearly as the rate of contraction increases. Amongst expanding establishments, the turnover rate appears largely flat at employment growth rates between 0 and 50 per cent but is decreasing at higher growth rates.

Table 3 reports the results from estimating Equation (2) with care worker turnover rate as the dependent variable. Column 1 is a linear version of the relationship presented in Figure 1A. The coefficients for positive and negative employment growth capture the cross-sectional correlation between turnover and employment growth amongst expanding and contracting establishments, respectively, while the constant term is the average turnover rate amongst establishments with stable employment. The estimates show that a one percentage point increase in rate of employment contraction is associated with a 0.90 percentage point rise in care worker turnover rate. Employment expansion, on the other hand, is not statistically related to turnover in this baseline case. The statistically significant constant implies that the average care establishment with no year-on-year change in care worker employment sees four out of 10 employees leave.

**Table 3:** Estimation results—turnover rate.

	**(1)**	**(2)**	**(3)**
Positive employment growth (i.e., expansion)	−0.049	−0.137^***^	−0.231^***^
	(0.033)	(0.030)	(0.023)
Negative employment growth (i.e., contraction)	−0.895^***^	−0.827^***^	−0.713^***^
	(0.027)	(0.025)	(0.025)
CQC (Overall) rating—Inadequate/Req improv.		−0.008	−0.002
		(0.016)	(0.013)
CQC (Overall) rating—No rating		0.024	0.012
		(0.025)	(0.019)
Two-year average total employment		−0.001^***^	−0.004^***^
		(0.000)	(0.001)
Average total employment—squared		0.000^**^	0.000^**^
		(0.000)	(0.000)
Direct care worker to service user ratio		0.000	−0.001
		(0.001)	(0.001)
Service users with dementia		0.073^***^	0.008
		(0.015)	(0.055)
Service users with mental infirmities		0.023	0.030
		(0.014)	(0.071)
Share of workers with dementia care training		0.088^***^	0.057
		(0.025)	(0.040)
Share of workers with DRPC training		0.044^*^	0.024
		(0.023)	(0.026)
Log (mean age of employees)		−0.637^***^	−0.089
		(0.076)	(0.120)
Log (mean experience of employees)		−0.040^**^	−0.022
		(0.016)	(0.030)
Log (mean hourly wage of care workers)		−0.082	−0.046
		(0.089)	(0.113)
Top quartile share of zero-hours contracts in sector		0.037^**^	0.006
		(0.019)	(0.020)
Manager/supervisor turnover rate (first lag)		0.280^***^	0.033^***^
		(0.019)	(0.011)
Unemployment rate at LAD-level		−0.042^***^	−0.005
		(0.013)	(0.014)
Log (mean hourly wage) of 1st quartile in LAD		−0.188	0.203
		(0.183)	(0.196)
Log (mean house price) at PCD-level		0.032	−0.027
		(0.028)	(0.081)
Care establishments HHI index at LAD-level		−0.991	−2.778
		(0.645)	(2.978)
Constant	0.424^***^	3.170^***^	1.004
	(0.008)	(0.606)	(1.180)
Year FE	No	Yes	Yes
Care Setting × Year FE	No	Yes	Yes
Sector × Year FE	No	Yes	Yes
Local Area × Year FE	No	Yes	Yes
Establishment FE	No	No	Yes
Observations	10,773	10,773	10,773
R-squared	0.095	0.285	0.863
Adj R-squared	0.0948	0.253	0.758

Robust standard errors clustered by Estab. ID in parentheses,

*** *p* < 0.01,

** *p* < 0.05,

* *p* < 0.1.

Column 2 reports the results from the specification including all covariates except for establishment-level fixed-effects. In this specification, the relationship between employment contraction and turnover is similar in sign and magnitude to Column 1, while the relationship between employment expansion and turnover is now negative and statistically significant. This change in magnitude and statistical significance suggests that systematic differences in turnover across sectors, care settings and other observable establishment characteristics mask some of the relationship between turnover and employment growth when we look at raw cross-sectional comparisons.

The coefficient estimates from Columns 1 and 2 reflect both the within-establishment relationship between turnover and employment growth and systematic differences across establishments. To isolate the within-establishment relationship, Column 3 adds to the previous specification establishment fixed effects. Controlling for time-invariant establishment-level heterogeneity via establishment fixed-effects leaves the turnover-employment growth relationship qualitatively unchanged but affects its magnitude. The estimates imply that a one percentage point increase in the employment contraction rate is associated with a 0.71 percentage point rise in turnover rate, while a one per cent increase in employment expansion rate is associated with a 0.23 percentage point fall in turnover rate.

To summarize, all three specifications point to a negative relationship between care worker turnover and employment along the entire growth distribution. Comparing across specifications suggests that part of this negative turnover-growth relationship is masked by systematic differences across establishments in both observable (comparing Column 1 with Column 2) and unobservable (Column 1 with Column 3) characteristics. Moreover, the estimates in Column 3 confirm that the negative association applies within establishments and is not an artifact of heterogeneity between establishments. In subsequent sections we integrate the above findings with our results from analyzing hiring to establish the role of staff retention in employment expansion.

#### Factors affecting turnover

Beyond the turnover-growth relationship, our results also shed light on other factors influencing care worker turnover amongst English care providers. Without controlling for establishment-level fixed-effects (Table 3, Column 2), we find statistically significant and positive coefficient estimates for having service users with dementia, the share of workers trained in dementia care and dignity in care, having a high share (top 25 per cent of sector) of care workers on zero-hours contracts, and turnover of managerial staff. We also find statistically significant negative coefficient estimates for (2 year) average total employment, mean age of employees, average employee years of experience and local area unemployment rate.

However, when controlling for establishment-level heterogeneity many of the relationships become statistically insignificant (Table 3, Column 3). On the one hand, this is to be expected as several variables, such as service user type, average employee age and years of experience, show little to no intertemporal variation within establishments and are hence “absorbed” by the establishment fixed-effects. On the other hand, the fact that coefficients on worker training and prevalence of zero-hours contracts become insignificant suggests that the cross-sectional relationship between these variables and turnover is in fact driven by establishment-level heterogeneity in contracting and training practices. Notably, the coefficient for managerial turnover remains significant at the one per cent level but its magnitude is almost ten times smaller. This suggests that the association between managerial staff stability and direct care staff turnover stems from both managerial staff turnover *per se* and unobserved heterogeneity between care establishments, with the latter having greater influence. Put differently, managerial staff instability and care worker turnover are related through two possible channels: directly, through insufficient supervision or mentorship, and indirectly, through persistent factors reflected by managerial staff stability, such as “organizational culture”. In this regard, our results suggest that while both channels of influence are active, the latter appears to account for a larger part of the association between managerial staff stability and care worker turnover.

### Hiring rates and employment growth

Figure 1B plots the non-parametric relationship between hiring and employment growth in the cross-section. It shows that new care worker hires as a share of employment is increasing over both negative and positive employment growth regions. The hiring-growth relationship is substantially less steep for contracting establishments and largely flat near the zero-growth region.

Table 4 reports the results from estimating Equation (2) with care worker hiring rate as the dependent variable. As before, the coefficient estimates in Column 1 capture the cross-sectional correlation between hiring and employment growth amongst expanding and contracting establishments, respectively. They imply that a one percentage point increase in employment expansion is associated with a 0.95 percentage point rise in hiring rate. In contrast, a one percentage point increase in employment contraction is associated with a 0.07 percentage point fall in care worker hiring rate. The estimated constant term, which represents the average hiring rate amongst zero-growth establishments, is almost identical to their average turnover rate (Table 3, Column 1). These two estimates together imply that the average care establishment with no year-on-year change in care worker employment sees four out of ten workers leave and replaces them one-for-one by the end of the year. Column 2 reports estimates after controlling for all covariates except for establishment fixed effects. Accounting for observable establishment characteristics reduces the magnitude of correlation between hiring and employment expansion but increases the magnitude of correlation between hiring and employment contraction.

**Table 4:** Estimation results—hiring rate.

	**(1)**	**(2)**	**(3)**
Positive employment growth (i.e., expansion)	0.947^***^	0.856^***^	0.760^***^
	(0.033)	(0.031)	(0.024)
Negative employment growth (i.e., contraction)	0.068^*^	0.141^***^	0.263^***^
	(0.035)	(0.032)	(0.030)
CQC (Overall) rating—Inadequate/Req improv.		−0.008	−0.000
		(0.017)	(0.014)
CQC (Overall) rating—No rating		0.023	0.013
		(0.026)	(0.021)
Two-year average total employment		−0.001^***^	−0.004^***^
		(0.000)	(0.001)
Average total employment—squared		0.000^**^	0.000^**^
		(0.000)	(0.000)
Direct care worker to service user ratio		0.000	−0.001
		(0.001)	(0.001)
Service users with dementia		0.076^***^	0.006
		(0.016)	(0.056)
Service users with mental infirmities (ex. MHA)		0.022	0.027
		(0.015)	(0.073)
Share of workers with dementia care training		0.083^***^	0.048
		(0.027)	(0.044)
Share of workers with DRPC training		0.044^*^	0.021
		(0.024)	(0.028)
Log (mean age of employees)		−0.651^***^	−0.060
		(0.080)	(0.136)
Log (mean experience of employees)		−0.040^**^	−0.037
		(0.018)	(0.037)
Log (mean hourly wage of care workers)		−0.083	−0.062
		(0.094)	(0.121)
Top quartile share of zero-hours contracts in sector		0.035^*^	0.003
		(0.020)	(0.020)
Manager/supervisor turnover rate (first lag)		0.300^***^	0.038^***^
		(0.021)	(0.012)
Unemployment rate at LAD-level		−0.045^***^	−0.009
		(0.014)	(0.015)
Log (mean hourly wage) of 1st quartile in LAD		−0.233	0.138
		(0.199)	(0.210)
Log (mean house price) at PCD-level		0.028	−0.038
		(0.030)	(0.085)
Care establishments HHI index at LAD-level		−1.155^*^	−2.698
		(0.678)	(3.062)
Constant	0.427^***^	3.402^***^	1.286
	(0.008)	(0.661)	(1.248)
Year FE	No	Yes	Yes
Care Setting × Year FE	No	Yes	Yes
Sector × Year FE	No	Yes	Yes
Local Area × Year FE	No	Yes	Yes
Establishment FE	No	No	Yes
Observations	10,773	10,773	10,773
R-squared	0.096	0.281	0.862
Adj R-squared	0.096	0.249	0.757

Robust standard errors clustered by Estab. ID in parentheses,

*** *p* < 0.01,

** *p* < 0.05,

* *p* < 0.1.

Column 3 adds establishment fixed effects to the specification in Column 2. This allows us to account for unobserved time-invariant heterogeneity across establishments. Furthermore, the coefficients on employment growth in this specification capture the within-establishment relationship between hiring and employment growth. The estimates imply that a one percentage point increase in employment growth is associated with a 0.76 percentage point rise in hiring rate, while a one percentage point increase in employment contraction is associated with a 0.26 percentage point fall in the hiring rate.

To summarize, the three specifications consistently show a positive relationship between care worker hiring and employment growth along the growth distribution. Additionally, the increasing magnitude of correlation between hiring and employment contraction suggests that part of the hiring-growth relationship is masked by systematic differences across establishments in both observable (comparing Column 1 with Column 2) and unobservable (Column 1 with Column 3) characteristics. Because each new hire for a contracting establishment is a replacement hire, our findings imply that establishments with more rapidly decreasing employment also tend to be the ones that are hiring replacements at a lower rate. In our subsequent analysis, we use information on the change in vacancies to understand if this relationship stems from active downsizing or difficulties in recruitment.

### The role of staff retention in employment growth

Our findings on the directions and magnitudes of the turnover-employment growth and hiring-employment growth relationships imply that measures which reduce the rate of staff turnover (i.e., staff retention policies), play an important role in employment growth. To see why, first note that the change in employment in a year is always equal to the difference between the number of hires and separations over that same period. Focusing on establishments with positive employment growth, this accounting relation means hiring net of separations must always be positive, but the hiring-growth and separations-growth relationships can be positive or negative in general.

Suppose that staff retention measures were irrelevant for employment growth. Then, we would expect to find either no systematic association between turnover and employment growth rates amongst expanding establishments, or that turnover rates increase with employment growth, as was found in Burgess et al. (28). Moreover, since employment growth must equal the inflow minus outflow of care workers, we would also expect hiring to increase at least one-for-one in employment growth.

In contrast, we consistently find a negative relationship between turnover and employment growth (Table 3) and positive but less than one-to-one relationship between hiring and employment growth (Table 4). These results imply that employment growth amongst LTC providers in England cannot be attributed solely to hiring behavior. Rather, the fact that higher employment growth is systematically associated with decreasing turnover within establishments suggests that better staff retention (and thereby reduced staff turnover) are also important for explaining workforce expansion.

**Figure 2:**
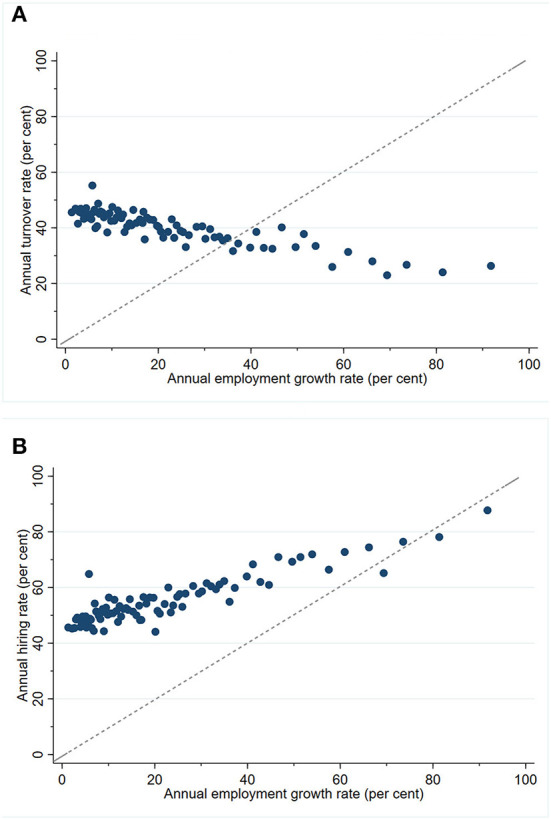
**(A)** Relationship between care worker turnover and employment growth (residualized means). **(B)** Relationship between care worker hiring and employment growth (residualized means).

The above arguments are summarized graphically in Figure 2, which plots the non-parametric relationship between hiring, turnover and employment growth, after accounting for establishment-level heterogeneity and calendar year effects. To obtain the figure, we first run regressions of hiring and turnover rates on establishment and year fixed effects, keep the residuals for each observation and add to each the corresponding mean turnover or hiring rate. These mean-adjusted residuals capture hiring and turnover behavior after excluding the systematic influence of time-invariant establishment-level heterogeneity and calendar year effects. Focusing on positive employment growth cases, we then plot the binned-scatter plots of residualized mean hiring and turnover rates against employment growth (as with Figure 1).

The key features to note are the downward-sloping locus of points in Panel (A) and the locus of points in Panel (B) which is upward-sloping but flatter than the reference 45-degree line. The former highlights the systematic negative relationship between turnover and employment growth within establishments. The latter shows that the hiring-employment growth relationship within establishments is positive but less than one-to-one.

### The role of recruitment frictions in replacement hiring

Our analysis of hiring rates showed that amongst establishments with employment contraction, higher rates of employment decline are associated with increased turnover rates and decreasing hiring rates. In this section, we will argue that recruitment difficulties can explain why hiring rates decrease amongst establishments with more rapid employment contraction. In general, it is possible that contracting establishments intentionally decrease replacement hiring as part of a downsizing policy. However, we argue that difficulties in recruitment (i.e., the inability to fill vacant positions) instead explain the observed relationship between replacement hiring and employment growth.

To make this argument, we use information on the annual change in vacancies. We focus on the change and not the stock of vacancies because the number of vacancies at any point in time consists of both previously existing vacancies that continue to be unfilled (i.e., persistent unmet labor demand) and/or newly posted vacancies (i.e., new labor demand). To disentangle the two factors we use the year-on-year change in the number of vacancies as our dependent variable and relate it to the change in employment (i.e., employment growth). To account for the confounding effect of new labor demand, we use the change in the number of people using an establishment's care services (hereafter “utilization”) as its proxy. Since each new vacancy represents an unfilled position (i.e., search for a new hire), if contracting employers were indeed intentionally reducing hiring to downsize, then, after controlling for changes in labor demand, we should not see a year-on-year change in vacancies after controlling for new labor demand.

**Figure 3:**
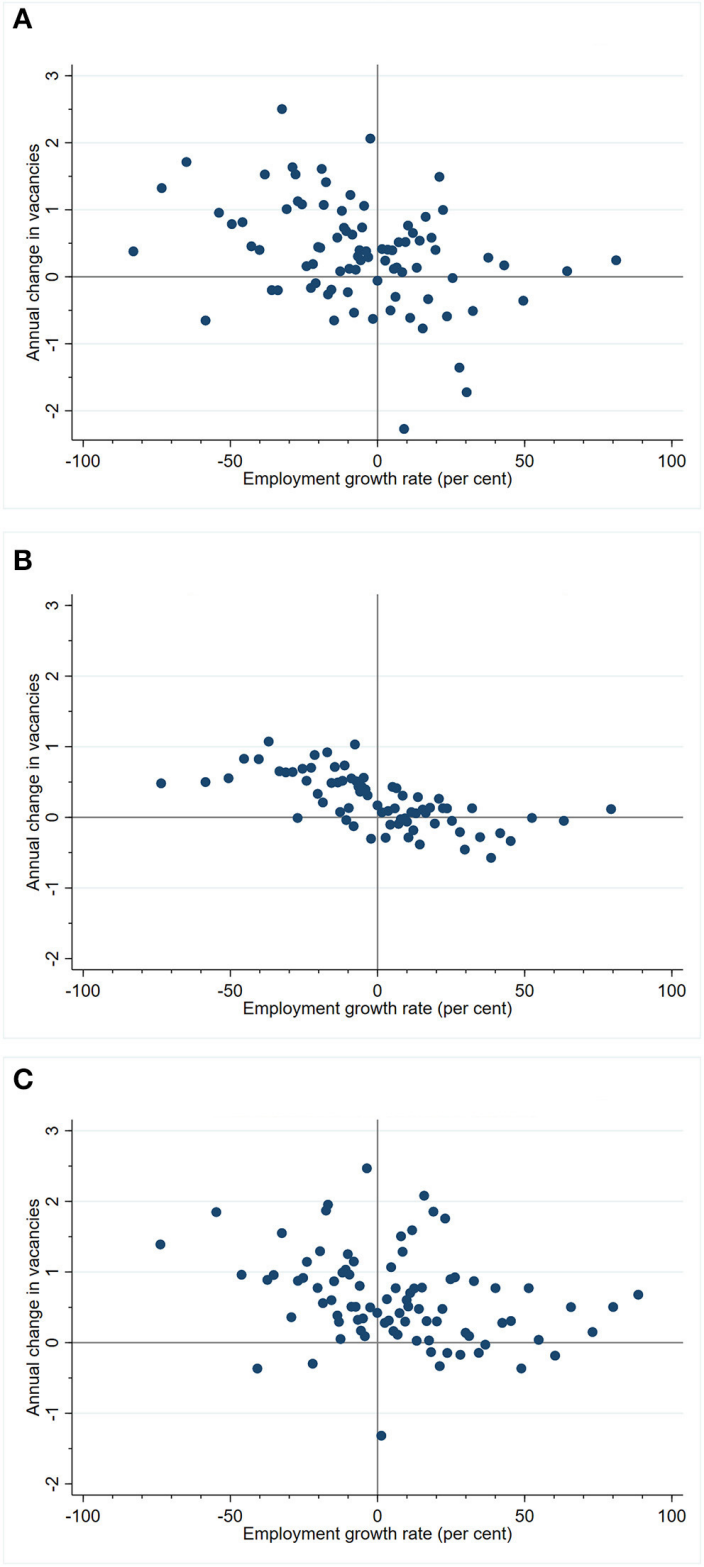
Relationship between unfilled vacancies and employment growth: **(A)** Establishments with decrease in service utilization. **(B)** Establishments with no change in service utilization. **(C)** Establishments with increase in service utilization.

Figure 3 presents descriptive evidence of the relationship between the annual change in vacancies and employment growth, after accounting for establishment-level heterogeneity and calendar year effects. To obtain this set of figures, we used the procedure described previously to obtain residualized measures of the change in vacancies for each observation. We then split the sample into three groups corresponding to establishments experiencing a decrease, no change or increase in utilization of their care services. Splitting the sample in this manner allows us to account roughly for the confounding effect of new labor demand. For each group, we plot a binned-scatter diagram (similar to Figures 1, 2) to capture the non-parametric relationship between the change in vacancies and employment growth.

There are two noteworthy features across all panels. First, the majority of all points in the negative employment growth region are in the north-west quadrant. Second, there is a negative association between the year-on-year change in unfilled care worker vacancies and employment growth.

The fact that most establishments with declining employment also report an annual rise in the number of vacancies, after accounting for growth in utilization of care services, is inconsistent with the hypothesis that these establishments are intentionally downsizing. Our argument is most evident in Figure 3B, which captures the case with no utilization growth and which shows that almost all establishments with declining employment report an increase in vacancies. Barring the possibility that only this group of contracting establishments anticipate a future jump in demand for services and hence labor demand, the remaining explanation is that the increase in year-on-year vacancies captures the inability of these establishments to replace employees who have left during the year.

To formalize the above intuition, Table 5 reports the results from estimating Equation (2) with the annual change in care worker vacancies as the dependent variable and additional piece-wise linear controls for positive and negative utilization growth. Our argument boils down to checking the sign of the coefficient on negative employment growth. In particular, the statistically significant negative coefficient on negative employment growth across all specifications implies that establishments with declining employment also systematically experience a year-on-year increase in vacancies. This finding, as we have argued, is inconsistent with the hypothesis that the observed decrease in replacement hiring amongst contracting establishments results from intentional downsizing.

**Table 5:** Estimation results—annual change in vacancies.

	**(1)**	**(2)**	**(3)**
Positive employment growth (i.e., expansion)	−0.503^**^	−0.583^**^	−0.852^**^
	(0.238)	(0.250)	(0.401)
Negative employment growth (i.e., contraction)	−1.072^***^	−0.840^***^	−1.145^***^
	(0.271)	(0.300)	(0.384)
Positive utilization growth	1.818^***^	1.694^***^	1.411^**^
	(0.442)	(0.417)	(0.595)
Negative utilization growth	−0.016	0.157	0.166
	(0.415)	(0.428)	(0.612)
CQC (Overall) rating—Inadequate/Req improv.		−0.058	−0.227
		(0.121)	(0.235)
CQC (Overall) rating—No rating		−0.190	−0.102
		(0.266)	(0.348)
Two-year average total employment		0.000	−0.029
		(0.004)	(0.034)
Average total employment—squared		0.000	0.000
		(0.000)	(0.000)
Direct care worker to service user ratio		−0.013^***^	0.018
		(0.005)	(0.022)
Service users with dementia		0.017	−0.116
		(0.077)	(0.539)
Service users with mental infirmities (ex. MHA)		0.078	2.672^***^
		(0.079)	(0.901)
Share of workers completed dementia care trg		0.305^*^	−0.204
		(0.167)	(0.452)
Share of workers completed DRPC trg		−0.241^*^	−0.245
		(0.144)	(0.245)
Log (mean age of employees)		1.373^***^	2.847^**^
		(0.422)	(1.413)
Log (mean experience of employees)		−0.504^***^	−0.931^**^
		(0.105)	(0.450)
Log (mean hourly wage of care workers)		0.795	0.670
		(0.611)	(1.978)
Top quartile share of zero-hours contracts in sector		−0.031	0.016
		(0.108)	(0.281)
Unemployment rate at LAD-level		0.073	−0.133
		(0.072)	(0.215)
Log (mean hourly wage) of 1st quartile in LAD		−0.274	−1.126
		(0.981)	(3.942)
Log (mean house price) at PCD-level		0.061	1.986
		(0.160)	(1.338)
Care establishments HHI index at LAD-level		2.891	−47.109
		(3.148)	(38.533)
Constant	0.154^***^	−6.201^*^	−31.151
	(0.049)	(3.240)	(20.728)
Year FE	No	Yes	Yes
Care Setting × Year FE	No	Yes	Yes
Sector × Year FE	No	Yes	Yes
Local Area × Year FE	No	Yes	Yes
Establishment FE	No	No	Yes
Observations	10,693	10,693	10,693
R-squared	0.006	0.099	0.360
Adj R-squared	0.06	0.06	−0.136

Robust standard errors clustered by Estab. ID in parentheses,

*** *p* < 0.01,

** *p* < 0.05,

* *p* < 0.1.

Regarding the magnitudes of our estimates, Column 1, which reports cross-sectional correlations while controlling for growth in utilization of care services, shows that a one percentage point increase in rate of employment contraction is associated with a 1.07 year-on-year rise unfilled vacancies. For expanding establishments, a one percentage point increase in the rate of employment expansion is instead associated with a 0.50 year-on-year decrease in unfilled vacancies. The statistically significant constant term implies that an establishment with stable employment and no change in number of care users has on average a 0.15 increase in year-on-year unfilled vacancies. Column 3, which controls for establishment fixed effects in addition to our battery of establishment-level characteristics, reports that within establishments a one percentage point increase in the rate of employment contraction is associated with an increase of 1.15 unfilled vacancies. In contrast, a one percentage point increase in the rate of employment expansion is associated with a decrease of 0.85 unfilled vacancies. We believe our findings are fairly robust to the issue of excess zeroes that is common in vacancy data. Intuitively, the logic of our argument rests on observing annual increases in vacancies amongst contracting establishments. To the extent that many establishments report zero change in vacancies, we would expect it to be more difficult to find evidence in favor of our argument. The fact that we have nevertheless found increases in vacancies thus points to the strength in support for our argument in the data.

The estimates of the relationship between employment contraction and change in vacancies are consistent with worsening recruitment difficulties amongst more rapidly contracting establishments. However, the current analysis is not designed to provide conclusive evidence and quantification of the link between employment decline and degree of recruitment frictions. The relationship between employment growth and change in vacancies amongst expanding establishments on the other hand highlights the role of vacancies as a recruitment device. That is, establishments with an increased need for labor report vacancies, which, via the recruitment process, lead to filling open positions.

Finally, we note that for given employment growth, growth in service utilization is positively associated with the change vacancies while a decrease in utilization is not statistically related to a change in vacancies. The positive and statistically significant estimate for growth in service utilization confirms our point regarding the confounding effect of new labor demand. In contrast, because vacancies cannot be used to reduce employment, there is unsurprisingly no link between the change in vacancies and the decreasing service utilization.

### Differences across sectors and care settings

To explore how the turnover, hiring and employment growth relationships may differ across sectors and care settings, we repeat the analysis using our preferred specification with establishment fixed for the separate sector and care setting subgroups. Table 6 summarizes the results from this subgroup analysis by reporting the estimates for the coefficients on employment growth and contractions.

**Table 6:** Estimation results—heterogeneity across sectors and care settings.

	**(1)**	**(2)**	**(3)**	**(4)**	**(5)**
	**Care setting**		**Sector**		
	***Residential***	***Domiciliary***	***Public***	***Private***	***Voluntary***
	**(A) Turnover rate**				
Positive employment growth (i.e., expansion)	−0.227^***^	−0.244^***^	−0.149^***^	−0.239^***^	−0.273^***^
	(0.028)	(0.041)	(0.036)	(0.027)	(0.084)
Negative employment growth (i.e., contraction)	−0.730^***^	−0.657^***^	−0.828^***^	−0.705^***^	−0.708^***^
	(0.031)	(0.047)	(0.062)	(0.031)	(0.054)
	**(B) Hiring rate**				
Positive employment growth (i.e., expansion)	0.767^***^	0.731^***^	0.851^***^	0.749^***^	0.723^***^
	(0.029)	(0.043)	(0.036)	(0.028)	(0.088)
Negative employment growth (i.e., contraction)	0.265^***^	0.299^***^	0.172^***^	0.264^***^	0.294^***^
	(0.033)	(0.055)	(0.062)	(0.037)	(0.055)
Year FE	Yes	Yes	Yes	Yes	Yes
Care Setting × Year FE	–	–	Yes	Yes	Yes
Sector × Year FE	Yes	Yes	–	–	–
Local Area × Year FE	Yes	Yes	Yes	Yes	Yes
Estab FE	Yes	Yes	Yes	Yes	Yes
Observations	7,958	2,751	571	8,289	1,727

Robust standard errors clustered by Estab. ID in parentheses,

*** *p* < 0.01,

**p < 0.05,

*p < 0.1.

#### Turnover and employment growth

Panel (A) of Table 6 reports the estimates for the regressions with turnover rates as the dependent variable. Comparing Columns 1 and 2, we find that the turnover-employment growth relationship is both qualitatively and quantitatively similar across residential and domiciliary care providers. Domiciliary care establishments with expanding employment tend to exhibit a slightly stronger negative relationship between turnover and employment growth compared to residential care establishments (−0.24 vs. −0.23). In contrast, domiciliary care providers with contracting employment show a slightly weaker relationship between employment decline and turnover relative to residential care (−0.66 vs. −0.73).

Differences across sectors are relatively larger (Columns 3 to 5). Amongst establishments with declining employment, the turnover-growth relationship is strongest amongst public sector care establishments (0.83), followed by voluntary sector (0.71) and then private sector establishments (0.70). Amongst establishments with growing employment, the turnover-growth relationship is strongest in the voluntary sector (0.27) followed by the private sector (0.24) and the public sector (0.15).

To assess if the differences described above are statistically significant, we estimate an extended version of Equation (2) in which the coefficients on positive and negative employment growth (i.e., *γ*^+^, *γ*^−^) are allowed to differ between sectors and care settings on the entire analysis sample. We then perform F-tests on the hypotheses $H_{0}:\gamma_{k}^{-}=\gamma_{l}^{-}$ vs. $H_{1}:\gamma_{k}^{-}\neq \gamma_{l}^{-}$, where *k, l* index different sectors or care settings and similarly for *γ*^+^. The estimates of the coefficients on employment growth from this auxiliary regression are reported in Columns 1 and 2 of Table A4 but we do not discuss them here, as they are both qualitatively and quantitatively very similar to the results already presented.

Across care settings, the F-tests do not reject the null of identical estimates of *γ*^+^ and *γ*^−^ for residential and domiciliary care establishments (*F* = 0.01, *p*-value = 0.93 for $H_{0}:\gamma_{res}^{+}=\gamma_{dom}^{+}$ and *F* = 0.72, *p*-value = 0.40 for $H_{0}:\gamma_{res}^{-}=\gamma_{dom}^{-}$). This implies that the strength of the turnover-employment growth relationship is similar across care settings. Comparing across sectors, we find that amongst establishments with increasing employment, the difference in the turnover-growth relationship is statistically significant between public and private sectors (*F* = 5.80, *p*-value = 0.016) and marginally significant between public and voluntary sectors (*F* = 3.41, *p*-value = 0.065). In contrast the turnover-growth relationship is not statistically different between private and voluntary sectors (*F* = 0.04, *p*-value 0.84). Amongst establishments with decreasing employment, the difference in the turnover-growth relationship is statistically significant between public and private sectors (*F* = 4.36, *p*-value = 0.04), marginally significant between public and voluntary sectors (*F* = 3.72, *p*-value = 0.05) and not significant between private and voluntary sectors (*F* = 0.01, *p*-value = 0.94).

#### Hiring and employment growth

Panel (B) of Table 6 reports the estimates for the regressions with hiring rates as the dependent variable. Columns 1 and 2 show that the hiring-employment growth relationship is similar across residential and domiciliary care providers. Residential care establishments with expanding workforces tend to show a slightly stronger positive relationship between hiring and employment growth compared to domiciliary care establishments (0.77 vs. 0.73). In contrast, residential care providers with contracting workforces show a slightly weaker relationship between employment decline and turnover relative to those in domiciliary care (0.27 vs. 0.30).

Columns 3 to 5 report the corresponding cross-sector differences. For expanding establishments, the estimates show that the hiring-growth relationship is strongest amongst public sector establishments (0.85), followed by those in the private (0.75) and voluntary (0.72) sectors. For establishments with declining employment, the hiring-growth relationship is strongest in the voluntary sector (0.29), followed by private (0.26) and then public sector (0.17).

To assess the statistical significance of these differences, we perform the auxiliary regression and statistical tests outlined in the previous section. The estimates, test statistics and *p*-values from this analysis are reported in Columns 3 and 4 of Table A4. Overall, we find that the hiring-employment growth relationship is not statistically different across care settings (*F* = 0.045, *p*-value = 0.832 for $H_{0}:\gamma_{res}^{+}=\gamma_{dom}^{+}\;$and *F* = 0.025, *p*-value = 0.875 for $H_{0}:\gamma_{res}^{-}=\gamma_{dom}^{-}$). Across sectors, the tests indicate that amongst establishments with increasing employment, the difference in hiring-growth relationship is statistically significant between public and private sectors (*F* = 5.802, *p*-value = 0.010) and marginally significant between public and voluntary sectors (*F* = 3.591, *p*-value = 0.058). In contrast, the hiring-growth relationship is not statistically different between private and voluntary sectors (*F* = 0.02, *p*-value 0.88). For establishments with decreasing employment, we find that the difference in hiring-growth relationship is marginally significant between public and voluntary sectors (*F* = 3.741, *p*-value = 0.053) and not significant between public and private sectors (*F* = 2.334, *p*-value = 0.127) and private and voluntary sectors (*F* = 0.444, *p*-value = 0.505).

#### Interpretation

Based on our arguments regarding the role of staff retention policies, the weaker association between turnover and employment growth in the public sector compared to private and voluntary sectors suggests that workforce expansion in the public sector is less sensitive to establishments' ability to retain workers. To the extent that the relationship between replacement hiring and employment decline reflects recruitment difficulties, the findings also imply that recruitment frictions have a greater impact on employment decline in the voluntary and private sector establishments compared to public sector establishments.

### Robustness of findings

In this section we explore robustness of our findings with respect to the on distribution of establishment sizes, functional form assumptions on the relationship between employment growth and worker flows and weighting to account for sample representativeness.

To explore how our findings may be affected when accounting for representativeness of the aggregate LTC sector, Table A3 reports results from repeating the fixed effects regression analyses reported in Tables 3–5 with our calibrated sampling weights. The idea behind weighting is to correct for under/over-representation in our sample along the dimensions used for calibration (details in the Note in Table A2). Comparing the weighted against unweighted estimates shows minor differences in the key estimates of interest. Given their similarity, our main analysis has opted for the more parsimonious approach of focusing on the unweighted analysis data.

To understand if our findings are sensitive to the distribution of establishment sizes in our data, Table A4 reports results from repeating the fixed effects regression analysis for subsamples split by establishment size. Panel (A) shows that the turnover-employment growth relationship is largely similar across establishment sizes. Similarly, Panel (B) shows that the estimates for the coefficients on employment growth in the hiring-employment growth regressions are largely similar across establishment size groups. This assures us that our results are not driven by the distribution of establishment sizes in our sample.

We next assess our assumptions on the functional form of *γ*(*g*), the relationship between employment growth and turnover/hiring. The top panel of Column 1 in Table A5 reproduces the estimates from Table 3 Column 3, displaying only the employment growth coefficients. The second panel reports the test statistic and *p*-value for a test of $H_{0}:\gamma^{+}=\gamma^{-}$. These reject the null of identical coefficients (*p*-value = 0.00) and indicate that a piece-wise functional form allowing for different turnover-growth relationships amongst contracting vs. growing establishments is consistent with the data. Similarly, Column 4 of Table A5 reproduces the estimates from Table 4 Column 3 and reports the test statistic and *p*-value for a test of difference in the coefficients on positive and negative employment growth. These reject the null of identical coefficients (*p*-value = 0.00) in support of allowing for different hiring-growth relationships amongst contracting vs. growing establishments.

The above tests for the appropriateness of the piece-wise specification (with a knot at zero growth) maintained the assumption of linearity outside of the knot. We next examine possible non-linearity in the turnover-employment growth and hiring-employment growth relationships while maintaining the assumption of a piece-wise form. We require the latter because the marginal effect of a change in employment growth rate on turnover/hiring rates for non-linear specifications depends on the reference employment growth value. As such, we cannot apply the direct test of a difference between marginal effects of positive vs. negative growth coefficients, as we did above.

Columns 2 and 3 of Table A5 report estimates of the coefficients on employment growth, allowing for separate quadratic (Column 2) and cubic (Column 3) functional forms for growing and contracting establishments. The coefficient estimates are all statistically significant at the one-percent level in Column 2 but are not significant at the 10-percent level for quadratic and cubic terms in Column 3. The statistically significant estimates for the quadratic specification in Column 2 indicate some curvature in the turnover-employment growth relationship. These estimates imply average marginal effects of employment growth on turnover equal to −0.289 for expanding establishments and −0.651 for contracting establishments. As these are close to the corresponding estimates of −0.231 (expanding establishments) and −0.713 (contracting establishments) for the linear specification, we believe the results from our preferred specification are reasonably robust to abstracting from this curvature in the turnover-employment growth relationship.

Similarly, Columns 5 and 6 of Table A5 report estimates from quadratic (Column 5) and cubic (Column 6) functional forms for the relationship between hiring and employment growth. The coefficient estimates in the quadratic specification are all statistically significant at the one-percent level, while all but one of the estimates for the coefficients on squared and cubed terms in Column 6 are statistically insignificant. Focussing on the quadratic form in Column 5, the estimates imply average marginal effects of employment growth on hiring equal to 0.687 for expanding establishments and 0.354 for contracting establishments. In comparison, the corresponding estimates from the piece-wise linear specification are 0.760 for expanding establishments and 0.263 for contracting establishments. As with the model for turnover, we believe that the benefit of having clear interpretations of the key coefficients on employment growth outweighs the relatively small bias from misspecification in the context of the present study.

## Discussion

In this study, we used a framework for analyzing the relationship between employment growth and worker flows and applied it to the context of care workers in the LTC sector in England. Using our estimates of the direction and magnitudes of these relationships, we shed light on the roles of staff retention and recruitment difficulties in the employment growth dynamics of LTC establishments.

Our finding that, amongst expanding establishments, turnover is decreasing with the rate of employment growth, and hiring rates are increasing but at a rate less than one-for-one differs from earlier studies (27, 28). It implies that unlike these cases, employment growth in our context is not driven solely by establishments' rate of hiring but also by their ability to control the outflow of existing workers. Put differently, this result highlights that staff retention policy is not only crucial for maintaining establishments' current workforce but is also important for achieving sustained workforce expansion. With respect to existing literature on turnover and retention in LTC, this finding introduces additional motivation, beyond care quality concerns, for improving retention amongst care staff.

To understand why the rate of replacement hiring is decreasing amongst contracting establishments, we made novel use of data on changes in vacancies. While standard “frictionless” models of labor markets would suggest that the observed slowdown in replacement hiring reflects intentional downsizing, our analysis found concurrent increases in unfilled vacancies which contradict this hypothesis. These findings instead suggest that difficulties in recruitment are important for explaining the pattern of decreasing hiring amongst contracting care establishments. While such recruitment difficulties have been suggested in workforce reports (1), the present study provides quantitative evidence of their presence and impact in the LTC sector.

Beyond employment growth, we find that unobserved establishment-level heterogeneity accounts for a large part of the cross-sectional variation in turnover and hiring rates. Nonetheless, our results confirm findings from previous studies that managers' turnover is positively related to care workers' turnover rate (5, 15, 21). In this respect, our analysis contributes to this discussion by highlighting that managerial staff turnover is related to care worker turnover both directly and indirectly, through the mediation of intangible organizational characteristics (i.e., culture). Moreover, our results show that the latter appears to account for a larger part of the association between managerial staff stability and care worker turnover.

While outside the scope of the present study, our analysis has uncovered a high level of staff churn, defined as the hiring and separations in excess of the levels required to achieve a given level of employment change, in the LTC labor market. Although not directly comparable, the average annual churn rate of about 81.8 per cent in our data is remarkably high compared to the average quarterly churn rate of about 22.8 per cent in the non-manufacturing sector in the U.S. (31). The literature on staff churn has interpreted churning as reflecting re-evaluation by workers and employers of the match between the worker and their job position. Based on this interpretation, care workers' employment conditions (e.g., low pay, lack of progression, competition for labor within the care sector and from outside the sector) and the nature of their work (significant amount of learning on the job, cognitively and emotionally challenging) are both likely contributors to the high rate of churn we observe.

### Policy implications

Our analysis suggests that policies that enable better staff retention and improve recruitment of new hires would aid in maintaining and growing the LTC workforce. Some measures, such as improving the terms of employment and increasing the possibility of career progression, are likely to aid in both of these aspects since these measures benefit existing employees and increase the attractiveness of care worker positions. The fact that we found turnover to be negatively related to employment growth also calls into question how best to expand the current LTC workforce to meet increasing demand. One approach is to increase hiring rates by “casting a wide net,” for example by relaxing selection criteria. However, this strategy may be counterproductive to the extent that it leads to hires with poorer job fit and subsequently higher rates of staff turnover. Alternatively, employers may consider longer-term job fit as an important criterion in recruitment. One example of such a strategy that has been used in practice is values-based recruitment (32). While this approach is likely to result in a slower rate of hiring, its long-term payoff is to reduce subsequent turnover, thus resulting in more sustainable employment expansion. Ultimately, the best approach depends on the extent and urgency of staffing shortfalls and is likely to differ between establishments.

### Limitations and future work

Throughout, we were careful to note that estimated relationships are associations and do not have causal interpretation. In general, hiring, separations and growth are likely to be linked through complex processes both within an organization and in the wider labor market. To examine these processes and establish causal links would require thorough structural equation modeling or exploit exogenous changes in labor market conditions, such as changes to the National Living Wage (the U.K. minimum wage). Also, our analysis excludes newly formed establishments due to the need to measure annual changes in employment. Nonetheless, this group may have different hiring and turnover dynamics and face different challenges compared to incumbent establishments. Understanding the relationship between turnover, hiring and employment growth for new care establishments is hence another potentially interesting area for future work.

As noted in our discussion, our analysis has also found high staff churn rates in the LTC sector (31). This suggests that there is a large amount of inefficient inflow and outflow of workers from LTC establishments and points to an urgent need to understand the source of such inefficiencies and the impact they may have on care provision. Relatedly, our study also highlights gaps in our current knowledge on the recruitment practices of LTC providers and the implications of these practices on staffing and quality of care. These are pertinent issues which similarly warrant further exploration.

## Data availability statement

The data analyzed in this study is subject to the following licenses/restrictions: The dataset is available from Skills for Care subject to a Data Sharing Agreement. Requests to access these datasets should be directed to analysis@skillsforcare.org.uk.

## Author contributions

HT contributed to the study conception, led the empirical analysis, and drafted the manuscript. FV contributed to the conception of the study, the empirical analysis, and the drafting of the manuscript. E-CS prepared the dataset, was involved in the early stages of the empirical analysis, and provided feedback on the manuscript. All authors contributed to the article and approved the submitted version.

## Funding

This study is part of the Retention and Sustainability of Social Care Workforce (RESSCW) project, funded by the Health Foundation's Efficiency Research Programme (AIMS ID 1325587). The Health Foundation is an independent charity committed to bringing about better health and health care for people in the UK.

## Conflict of interest

The authors declare that the research was conducted in the absence of any commercial or financial relationships that could be construed as a potential conflict of interest.

## Publisher's note

All claims expressed in this article are solely those of the authors and do not necessarily represent those of their affiliated organizations, or those of the publisher, the editors and the reviewers. Any product that may be evaluated in this article, or claim that may be made by its manufacturer, is not guaranteed or endorsed by the publisher.
