# Supplementary material for: Recruitment, retention and employment growth in the long-term care sector in England
Source: Front Public Health. 2022 Oct 28;10:969098. doi: 10.3389/fpubh.2022.969098 (PMC9650477; doi:10.3389/fpubh.2022.969098)
Supplement: Supplementary file 1 [file Data_Sheet_1.docx]

**Appendix**

**Table A1. Variable definitions**

| Variable | Definition |
| --- | --- |
| Two-year average total employment | **(***No. of employees in care worker job role in current year* **plus** *No. of employees in care worker job role in previous year***)** **divided by two** |
| Turnover rate of care workers | *No. of employees in care worker job role that left in the previous 12 months* **divided by** *Two-year average total employment* |
| Hiring rate of care workers | *No. of employees in care worker job role that started in the previous 12 months* **divided by** *Two-year average total employment* |
| Vacancy rate of care workers | *No. of care worker job role vacancies at the time of update* in current year **divided by (***No. of care worker job role vacancies at the time of update* in current year ***plus*** *Two-year average total employment***)** |
| Care worker employment growth rate | **(***No. of employees in care worker job role in current year* **minus** *No. of employees in care worker job role in previous year***)** **divided by** *Two-year average total employment* |
| Annual change in care worker vacancies | *No. of care worker job role vacancies at the time of update* in current year **minus** *No. of care worker job role vacancies at the time of update* in previous year |
| Service utilisation growth rate | (*Uptake of care service at the time of update in current year* **minus** *Uptake of care service at the time of update in previous year*) **divided by** Two-year average utilisation |
| Direct care worker to service user ratio | *No. of employees in direct care* *providing job roles in current year* **divided by** *Uptake of service at the time of update in current year* |
| Turnover rate of managers/supervisors | *No. of employees in managerial/supervisory job roles that left in the previous 12 months* **divided by** Two-year average number of staff *in managerial/supervisory job roles* |
| Service users with dementia | **Dummy variable** for any *services users with dementia* |
| Service users with mental infirmities (ex. MHA) | **Dummy variable** for any *services users with mental disorders or infirmities, excluding dementia and service users with autistic spectrum disorder* |
| Share of workers completed dementia care trg | *Count of employees in care worker job role who have completed Dementia care training* **divided by** *Count of employees in care worker job role* |
| Share of workers completed DRPC trg | *Count of employees in care worker job role who have completed Dignity, Respect, Person Centred care training* **divided by** *Count of employees in care worker job role* |
| Mean age of employees | Mean *age of all staff on records in current year* |
| Mean years of experience of employees | Mean *experience of all staff on records in current year*, where experience equals *current year* **minus** *year staff started working in social care sector* |
| Mean hourly wage of employed care workers | Mean *hourly wage of* *all of permanent and temporary staff in care worker job role* |
| Share of care workers on zero-hours contracts | *Count of employees in care worker job role employed on zero-hours contract* **divided by** *employees in care worker job role* |
| Top quartile share of zero-hours contracts in sector | **Dummy variable** for *share of care workers on zero-hours contracts in top quartile of establishments in the same sector* |
| CQC (Overall) rating - Inadequate/Req improv | **Dummy variable** for *CQC (Overall) rating of Inadequate or Requires Improvement* |
| CQC (Overall) rating - Good/Outstanding | **Dummy variable** for *CQC (Overall) rating of Good or Outstanding* |
| Unemployment rate at LAD-level | *Unemployment rate at Local Authority District level* |
| Log(mean hourly wage) of 1st quartile in LAD | **Log of the mean of** *hourly wages in the first quartile at Local Authority District level* |
| Log(mean house price) at PCD-level | **Log of the mean of** *house prices at Postcode District level* |
| Care establishments HHI index at LAD-level | *Herfindahl-Hirschman Index for care establishments at Local Authority District level* |
| Note 1: Employee is defined as permanent or temporary staff on record.  Note 2: Care service is either Care home services with nursing, Care home services without nursing or Domiciliary care services. | |

**Table A2. Weighted and unweighted summary statistics**

|  | Unweighted | | Weighted | |
| --- | --- | --- | --- | --- |
| Variable | Mean | Std Dev. | Mean | Std Dev. |
| Turnover rate of care workers | 0.503 | 0.517 | 0.539 | 0.543 |
| Hiring rate of care workers | 0.503 | 0.547 | 0.538 | 0.576 |
| Annual change in care worker vacancies | 0.282 | 4.491 | 0.306 | 5.010 |
| Care worker employment growth rate | -0.006 | 0.279 | -0.008 | 0.300 |
| Positive employment growth | 0.391 | 0.488 | 0.391 | 0.488 |
| Negative employment growth | 0.426 | 0.494 | 0.431 | 0.495 |
| Service utilisation growth rate | 0.009 | 0.211 | 0.006 | 0.220 |
| Two-year average total employment | 47.634 | 47.815 | 49.566 | 50.801 |
| Direct care worker to service user ratio | 1.665 | 4.250 | 1.686 | 4.457 |
| Service users with dementia | 0.554 | 0.497 | 0.571 | 0.495 |
| Service users with mental infirmities (ex. MHA) | 0.643 | 0.479 | 0.678 | 0.467 |
| Share of workers completed dementia care trg | 0.258 | 0.327 | 0.269 | 0.331 |
| Share of workers completed DRPC trg | 0.187 | 0.309 | 0.196 | 0.315 |
| Mean age of employees | 43.182 | 4.753 | 43.129 | 4.788 |
| Mean years of experience of employees | 8.811 | 3.893 | 8.517 | 3.863 |
| Mean hourly wage of employed care workers | 7.761 | 0.756 | 7.753 | 0.709 |
| Share of care workers on zero-hours contracts | 0.171 | 0.333 | 0.237 | 0.383 |
| Turnover rate of managers/supervisors | 0.320 | 0.499 | 0.337 | 0.512 |
| CQC (Overall) rating - Inadequate/Req improv. | 0.135 | 0.341 | 0.158 | 0.364 |
| CQC (Overall) rating - Good/Outstanding | 0.827 | 0.378 | 0.716 | 0.451 |
| CQC (Overall) rating - No rating | 0.039 | 0.193 | 0.126 | 0.332 |
| Residential care | 0.741 | 0.438 | 0.642 | 0.479 |
| Domiciliary care | 0.259 | 0.438 | 0.358 | 0.479 |
| Public sector | 0.061 | 0.239 | 0.029 | 0.167 |
| Private sector | 0.770 | 0.421 | 0.837 | 0.369 |
| Voluntary sector | 0.169 | 0.375 | 0.134 | 0.341 |
| Observations | 10,773 | | | |
| Note: Weights from calibration by raking procedure, with year x care setting target totals obtained from CQC registry. Calibration variables for residential care are sector, main service type (nursing / non-nursing care), capacity, CQC Overall rating and region. Calibration variables for domiciliary care are sector, CQC Overall rating and region. | | | | |

**Table A3. Weighted and unweighted regression results**

|  | (1) | (2) | (3) | | (4) | (5) | (6) |
| --- | --- | --- | --- | --- | --- | --- | --- |
|  | Care worker turnover rate | Care worker hiring rate | Change in vacancies | | Care worker turnover rate | Care worker hiring rate | Change in vacancies |
|  | *Weighted* | | | | *Unweighted* | | |
| Positive employment growth (i.e. expansion) | -0.220*** | 0.768*** | -0.848* | | -0.231*** | 0.760*** | -0.852** |
|  | (0.026) | (0.026) | (0.489) | | (0.023) | (0.024) | (0.401) |
| Negative employment growth (i.e. contraction) | -0.725*** | 0.238*** | -1.337*** | | -0.713*** | 0.263*** | -1.145*** |
|  | (0.027) | (0.035) | (0.407) | | (0.025) | (0.030) | (0.384) |
| CQC (Overall) rating - Inadequate/Req improv. | 0.005 | 0.007 | -0.215 | | -0.002 | -0.000 | -0.227 |
|  | (0.014) | (0.015) | (0.258) | | (0.013) | (0.014) | (0.235) |
| CQC (Overall) rating - No rating | 0.006 | 0.012 | -0.149 | | 0.012 | 0.013 | -0.102 |
|  | (0.020) | (0.022) | (0.350) | | (0.019) | (0.021) | (0.348) |
| Two-year average total employment | -0.004*** | -0.005*** | -0.039 | | -0.004*** | -0.004*** | -0.029 |
|  | (0.001) | (0.001) | (0.028) | | (0.001) | (0.001) | (0.034) |
| Average total employment - squared | 0.000** | 0.000** | 0.000 | | 0.000** | 0.000** | 0.000 |
|  | (0.000) | (0.000) | (0.000) | | (0.000) | (0.000) | (0.000) |
| Direct care worker to service user ratio | -0.001 | -0.000 | 0.031 | | -0.001 | -0.001 | 0.018 |
|  | (0.001) | (0.002) | (0.023) | | (0.001) | (0.001) | (0.022) |
| Service users with dementia | 0.004 | 0.007 | -0.083 | | 0.008 | 0.006 | -0.116 |
|  | (0.056) | (0.057) | (0.567) | | (0.055) | (0.056) | (0.539) |
| Service users with mental infirmities (ex. MHA) | 0.033 | 0.016 | 2.196*** | | 0.030 | 0.027 | 2.672*** |
|  | (0.075) | (0.078) | (0.785) | | (0.071) | (0.073) | (0.901) |
| Share of workers with dementia care training | 0.081* | 0.069 | -0.554 | | 0.057 | 0.048 | -0.204 |
|  | (0.044) | (0.049) | (0.561) | | (0.040) | (0.044) | (0.452) |
| Share of workers with DRPC training | 0.019 | 0.017 | 0.008 | | 0.024 | 0.021 | -0.245 |
|  | (0.028) | (0.031) | (0.268) | | (0.026) | (0.028) | (0.245) |
| Log(mean age of employees) | -0.055 | 0.022 | 3.922** | | -0.089 | -0.060 | 2.847** |
|  | (0.136) | (0.166) | (1.686) | | (0.120) | (0.136) | (1.413) |
| Log(mean experience of employees) | -0.016 | -0.042 | -1.089** | | -0.022 | -0.037 | -0.931** |
|  | (0.034) | (0.045) | (0.502) | | (0.030) | (0.037) | (0.450) |
| Log(mean hourly wage of care workers) | -0.071 | -0.097 | 2.682 | | -0.046 | -0.062 | 0.670 |
|  | (0.125) | (0.139) | (2.173) | | (0.113) | (0.121) | (1.978) |
| Top quartile share of zero-hours contracts in sector | 0.016 | 0.014 | 0.109 | | 0.006 | 0.003 | 0.016 |
|  | (0.021) | (0.022) | (0.326) | | (0.020) | (0.020) | (0.281) |
| Manager/supervisor turnover rate (first lag) | 0.038*** | 0.045*** |  | | 0.033*** | 0.038*** |  |
|  | (0.013) | (0.014) |  | | (0.011) | (0.012) |  |
| Unemployment rate at LAD-level | -0.003 | -0.007 | -0.109 | | -0.005 | -0.009 | -0.133 |
|  | (0.016) | (0.017) | (0.257) | | (0.014) | (0.015) | (0.215) |
| Log(mean hourly wage) of 1st quartile in LAD | 0.253 | 0.185 | -1.393 | | 0.203 | 0.138 | -1.126 |
|  | (0.227) | (0.243) | (4.973) | | (0.196) | (0.210) | (3.942) |
| Log(mean house price) at PCD-level | -0.037 | -0.045 | 0.429 | | -0.027 | -0.038 | 1.986 |
|  | (0.090) | (0.095) | (1.494) | | (0.081) | (0.085) | (1.338) |
| Care establishments HHI index at LAD-level | -4.439 | -4.575 | -39.527 | | -2.778 | -2.698 | -47.109 |
|  | (3.601) | (3.774) | (46.603) | | (2.978) | (3.062) | (38.533) |
| Positive utilisation growth |  |  | 1.476* | |  |  | 1.411** |
|  |  |  | (0.804) | |  |  | (0.595) |
| Negative utilisation growth |  |  | 0.684 | |  |  | 0.166 |
|  |  |  | (0.594) | |  |  | (0.612) |
| Constant | 0.982 | 1.117 | -18.981 | | 1.004 | 1.286 | -31.151 |
|  | (1.319) | (1.403) | (24.241) | | (1.180) | (1.248) | (20.728) |
|  |  |  |  | |  |  |  |
| Year FE | Yes | Yes | Yes | | Yes | Yes | Yes |
| Care Setting x Year FE | Yes | Yes | Yes | | Yes | Yes | Yes |
| Sector x Year FE | Yes | Yes | Yes | | Yes | Yes | Yes |
| Local Area x Year FE | Yes | Yes | Yes | | Yes | Yes | Yes |
| Estab FE | Yes | Yes | Yes | | Yes | Yes | Yes |
|  |  |  |  | |  |  |  |
| Observations | 10,773 | 10,773 | 10,693 | | 10,773 | 10,773 | 10,693 |
| R-squared | 0.874 | 0.872 | 0.454 | | 0.863 | 0.862 | 0.360 |
| Adj R-squared | 0.779 | 0.775 | 0.0315 | | 0.758 | 0.757 | -0.136 |
| Robust standard errors clustered by Estab. ID in parentheses, *** p<0.01, ** p<0.05, * p<0.1 | | | |  |  |  |  |

**Table A4. Checks for heterogeneity across establishment size**

|  | Two-year average total employment | | | |
| --- | --- | --- | --- | --- |
|  | <10 | 10-49 | 50-99 | 100+ |
|  | (1) | (2) | (3) | (4) |
|  | (A) Turnover rate | | | |
| Employment growth (positive growth) | -0.0823 | -0.272*** | -0.217*** | -0.220*** |
|  | (0.0731) | (0.0479) | (0.0578) | (0.0604) |
| Employment growth (negative growth) | -0.816*** | -0.698*** | -0.671*** | -0.722*** |
|  | (0.0945) | (0.0351) | (0.0574) | (0.0581) |
|  | (B) Hiring rate | | | |
| Employment growth (positive growth) | 0.894*** | 0.700*** | 0.776*** | 0.801*** |
|  | (0.0773) | (0.0431) | (0.0588) | (0.0604) |
| Employment growth (negative growth) | 0.177 | 0.299*** | 0.334*** | 0.234*** |
|  | (0.111) | (0.0361) | (0.0596) | (0.0612) |
| Year FE | Yes | Yes | Yes | Yes |
| Care Setting x Year FE | Yes | Yes | Yes | Yes |
| Sector x Year FE | Yes | Yes | Yes | Yes |
| Local Area x Year FE | Yes | Yes | Yes | Yes |
| Estab FE | Yes | Yes | Yes | Yes |
| Observations | 538 | 7,133 | 2,595 | 818 |

**Table A5. Auxiliary regressions and tests for robustness to functional form assumptions**

|  | (1) | (2) | (3) | | (4) | (5) | (6) |
| --- | --- | --- | --- | --- | --- | --- | --- |
|  | Care worker turnover rate | Care worker turnover rate | Care worker turnover rate | | Care worker hiring rate | Care worker hiring rate | Care worker hiring rate |
| Positive employment growth | -0.231*** | -0.346*** | -0.344*** | | 0.760*** | 0.620*** | 0.661*** |
|  | (0.023) | (0.043) | (0.068) | | (0.024) | (0.046) | (0.070) |
| Positive employment growth -squared |  | 0.128*** | 0.138 | |  | 0.151*** | 0.086 |
|  |  | (0.040) | (0.161) | |  | (0.042) | (0.164) |
| Positive employment growth -cubed |  |  | -0.008 | |  |  | 0.027 |
|  |  |  | (0.084) | |  |  | (0.085) |
| Negative employment growth | -0.713*** | -0.591*** | -0.637*** | | 0.263*** | 0.445*** | 0.275*** |
|  | (0.025) | (0.040) | (0.074) | | (0.030) | (0.054) | (0.091) |
| Negative employment growth -squared |  | 0.136*** | 0.006 | |  | 0.208*** | -0.267 |
|  |  | (0.039) | (0.186) | |  | (0.064) | (0.254) |
| Negative employment growth -cubed |  |  | -0.069 | |  |  | -0.250* |
|  |  |  | (0.090) | |  |  | (0.140) |
| $H_{0}:\gamma^{+}$=$\gamma^{-}$: |  |  |  | |  |  |  |
| *F(1,4198)* | 140.195 |  |  | | 115.996 |  |  |
| p-value | 0.000 |  |  | | 0.000 |  |  |
|  |  |  |  | |  |  |  |
| Covariates from full regression model | Yes | Yes | Yes | | Yes | Yes | Yes |
| Year FE | Yes | Yes | Yes | | Yes | Yes | Yes |
| Care Setting x Year FE | Yes | Yes | Yes | | Yes | Yes | Yes |
| Sector x Year FE | Yes | Yes | Yes | | Yes | Yes | Yes |
| Local Area x Year FE | Yes | Yes | Yes | | Yes | Yes | Yes |
| Estab FE | Yes | Yes | Yes | | Yes | Yes | Yes |
|  |  |  |  | |  |  |  |
| Observations | 15,613 | 15,613 | 15,613 | | 15,613 | 15,613 | 15,613 |
| R-squared | 0.844 | 0.845 | 0.845 | | 0.843 | 0.844 | 0.844 |
| Adj R-squared | 0.751 | 0.752 | 0.752 | | 0.749 | 0.750 | 0.750 |
| Robust standard errors clustered by Estab. ID in parentheses, *** p<0.01, ** p<0.05, * p<0.1 | | | |  |  |  |  |

**Table A6. Auxiliary regressions and tests for heterogeneous employment growth coefficients**

|  | (1) | (2) | (3) | (4) |
| --- | --- | --- | --- | --- |
| VARIABLES | Care worker turnover rate | Care worker turnover rate | Care worker hiring rate | Care worker hiring rate |
| Positive employment growth x Public sector | -0.107** |  | 0.896*** |  |
|  | (0.049) |  | (0.050) |  |
| Positive employment growth x Private sector | -0.240*** |  | 0.749*** |  |
|  | (0.026) |  | (0.028) |  |
| Positive employment growth x Voluntary sector | -0.254*** |  | 0.738*** |  |
|  | (0.064) |  | (0.067) |  |
| Negative employment growth x Public sector | -0.846*** |  | 0.155** |  |
|  | (0.060) |  | (0.061) |  |
| Negative employment growth x Private sector | -0.705*** |  | 0.263*** |  |
|  | (0.030) |  | (0.036) |  |
| Negative employment growth x Voluntary sector | -0.701*** |  | 0.303*** |  |
|  | (0.047) |  | (0.048) |  |
| Positive employment growth x Residential |  | -0.229*** |  | 0.763*** |
|  |  | (0.028) |  | (0.029) |
| Positive employment growth x Domiciliary |  | -0.233*** |  | 0.753*** |
|  |  | (0.039) |  | (0.042) |
| Negative employment growth x Residential |  | -0.728*** |  | 0.267*** |
|  |  | (0.030) |  | (0.033) |
| Negative employment growth x Domiciliary |  | -0.683*** |  | 0.256*** |
|  |  | (0.043) |  | (0.060) |
| $H_{0}:\gamma_{Public}^{+}$=$\gamma_{Private}^{+}$: |  |  |  |  |
| F(1,4198) | 5.802 |  | 5.802 |  |
| p-value | 0.016 |  | 0.010 |  |
| $H_{0}:\gamma_{Public}^{+}$=$\gamma_{Voluntary}^{+}$ |  |  |  |  |
| F(1,4198) | 3.409 |  | 3.591 |  |
| p-value | 0.065 |  | 0.058 |  |
| $H_{0}:\gamma_{Private}^{+}$=$\gamma_{Voluntary}^{+}$ |  |  |  |  |
| F(1,4198) | 0.044 |  | 0.022 |  |
| p-value | 0.835 |  | 0.881 |  |
| $H_{0}:\gamma_{Public}^{-}$=$\gamma_{Private}^{-}$: |  |  |  |  |
| F(1,4198) | 4.357 |  | 2.334 |  |
| p-value | 0.037 |  | 0.127 |  |
| $H_{0}:\gamma_{Public}^{-}$=$\gamma_{Voluntary}^{-}$ |  |  |  |  |
| F(1,4198) | 3.721 |  | 3.741 |  |
| p-value | 0.054 |  | 0.053 |  |
| $H_{0}:\gamma_{Private}^{-}$=$\gamma_{Voluntary}^{-}$ |  |  |  |  |
| F(1,4198) | 0.006 |  | 0.444 |  |
| p-value | 0.938 |  | 0.505 |  |
| $H_{0}:\gamma_{Residential}^{+}$=$\gamma_{Domiciliary}^{+}$ |  |  |  |  |
| F(1,4198) |  | 0.007 |  | 0.045 |
| p-value |  | 0.934 |  | 0.832 |
| $H_{0}:\gamma_{Residential}^{-}$=$\gamma_{Domiciliary}^{-}$ |  |  |  |  |
| F(1,4198) |  | 0.719 |  | 0.025 |
| p-value |  | 0.397 |  | 0.875 |
| Year FE | Yes | Yes | Yes | Yes |
| Care Setting x Year FE | Yes | Yes | Yes | Yes |
| Sector x Year FE | Yes | Yes | Yes | Yes |
| Local Area x Year FE | Yes | Yes | Yes | Yes |
| Estab FE | Yes | Yes | Yes | Yes |
| Observations | 10,773 | 10,773 | 10,773 | 10,773 |
